# Supplementary material for: Direct Synthesis of Oxynitride Nanowires through Atmospheric Pressure Chemical Vapor Deposition
Source: Nanomaterials (Basel). 2020 Dec 14;10(12):2507. doi: 10.3390/nano10122507 (PMC7764907; doi:10.3390/nano10122507)
Supplement: Supplementary file 1 [file nanomaterials-10-02507-s001.pdf]

Supplementary information

Supplementary information 1: Atmospheric chemical vapor deposition apparatus and the implemented adjustments.

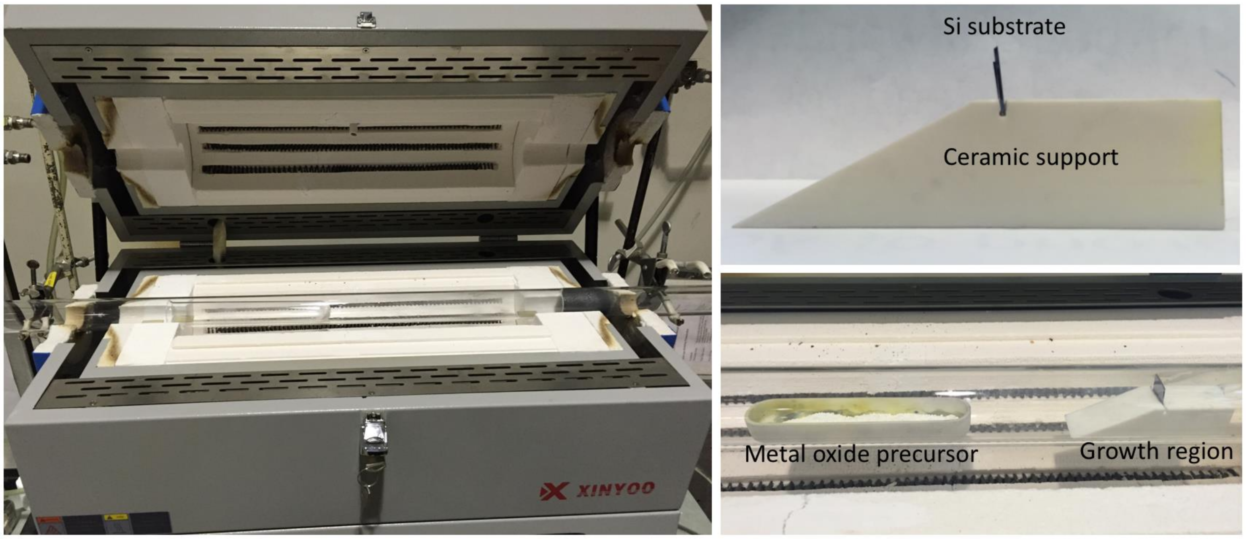

Figure 1: The CVD apparatus used for the synthesis of GaN:ZnO solid solution nanowires. Modifications to enhance the local mass transfer flux of vapor deposition through positioning the Si substrate on the ceramic support.

Supplementary information 2: Atmospheric chemical vapor deposition of GaN:ZnO with horizontal substrate.

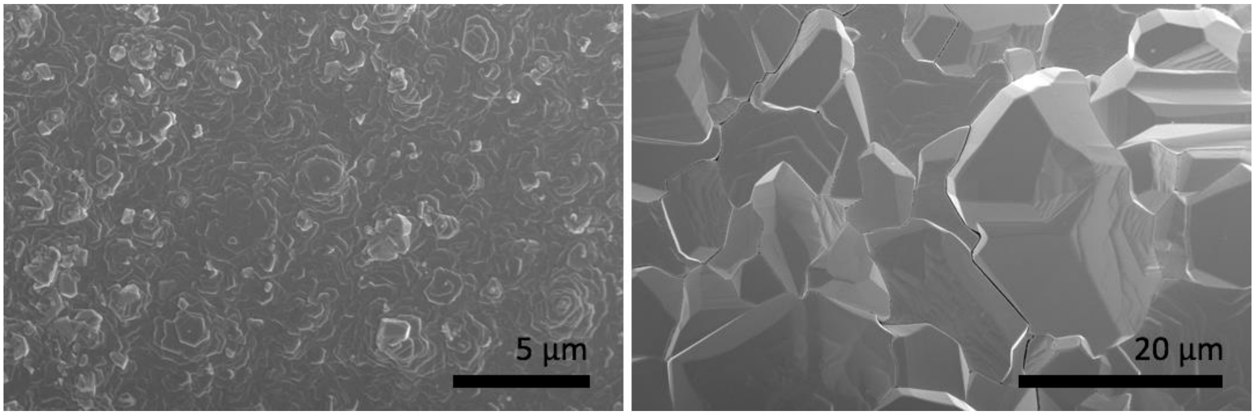

Figure 2: SEM image of the substrate placed horizontally at Ts = 1,000 °C for 30 min at D = 8 cm downstream of the Ga_2O_3 and ZnO source materials, where uniform growth of nanowires was not observed. Left micrograph shows the substrate positioned face-up, and right image displays the substrate positioned face-down. In some occasions, 1-D nanostructures with low aspect ratio were observed on the corners and edges of the substrate.

Supplementary information 3: Surface growth via non-epitaxial approach

To explore the role of substrate surface crystallography, Au-coated amorphous quartz substrates were used for the growth of GaN:ZnO thin film through non-epitaxial atmospheric pressure chemical vapor deposition (APCVD).

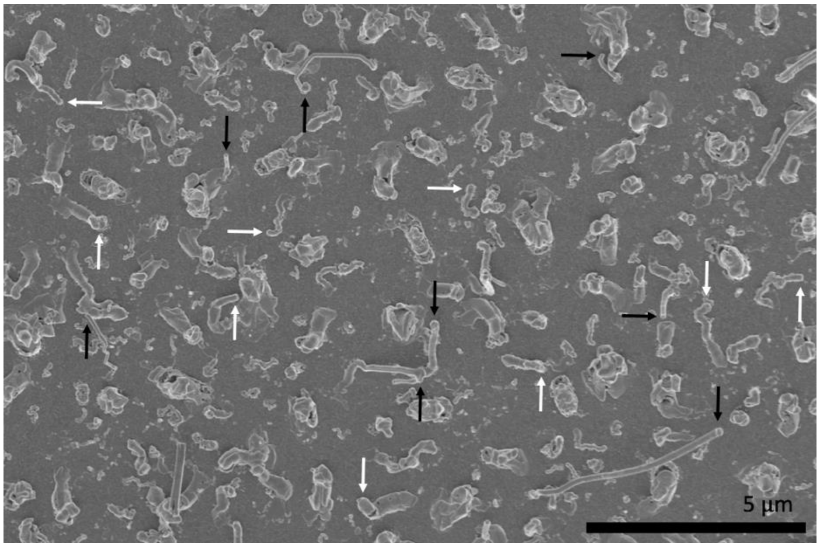

Figure 3: SEM image of a quartz substrate processed at Ts = 1000 °C for t = 30 min and D = 8 cm downstream of the Ga_2O_3 and ZnO source materials. Two distinct types of nanostructures were observed on the substrate, denoted with black and white arrows, black arrows showing those nanowires that reached complete growth, and white arrows indicating those grown via surface growth (crawling).

Depending on the distance of the substrates from the center of the heated zone (D), the surface of the substrate was coated with various colors, implying the deposition of phases with distinct chemistries and morphologies. The scanning electron microscopy (SEM) image of the processed quartz substrate in Figure 3 indicates that the fabrication

of nanowire arrays at high density cannot be realized through the atmospheric pressure non-epitaxial route. It is generally accepted that to form well-defined crystals, a reversible pathway between building blocks on the surface of the solid and those inside the fluid phase must exist. This allows building blocks to maintain their positions according to the existing crystal lattice [1]. A lack of such crystalline support (non-epitaxial) results in random and slow growth of 1-D nanostructures rather than dense and defined structures. Due to a lack of suitable crystalline sites on the surface of the quartz substrate during the APCVD growth, as marked with white arrows in **Figure 3**, Au catalyst NPs migrated on the substrate surface and formed horizontal nanowires (surface growth) until they reached a crystalline deposit with matching lattice dimensions (*e.g.*, deposited ZnO, GaN, or their solid solutions). This growth mechanism, referred to as "crawling," occurs when epitaxial growth conditions cannot be satisfied. Thus, these nanowires could not reach complete growth with the given synthesis duration. The growth of nanowires parallel to the substrate surface is actually very useful due to their potential for optoelectronic device fabrication and precise placement of single nanowires [2]. As indicated by the black arrows in **Figure 3**, the synthesized nanowires are capped with an Au NP, confirming the VLS growth mechanism. The average diameter of those nanowires reached complete growth between 100–120 nm, with a length of 1.7–5.1 μm. The areal density of the nanowires on the surface varied over a wide range, reaching 7.0×10^{6} cm^{-2} in some regions.

Supplementary information 4: Exposed crystallographic facets and growth direction

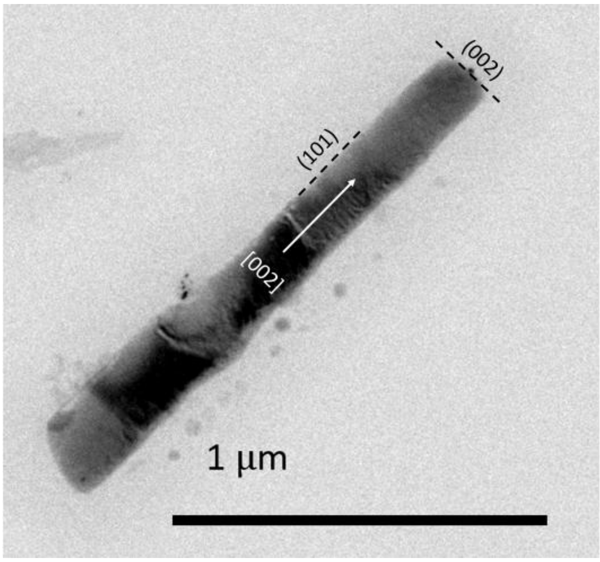

Figure 4: Low resolution scanning transmission electron microscopy (STEM) image of nanowires prepared at 1000 °C for 30 min at D = 8 cm showing the growth direction, along the facets on the side and tip.

Supplementary information 5: Photoluminescence (PL) analysis of synthesized GaN:ZnO nanowires and GaN:ZnO powder.

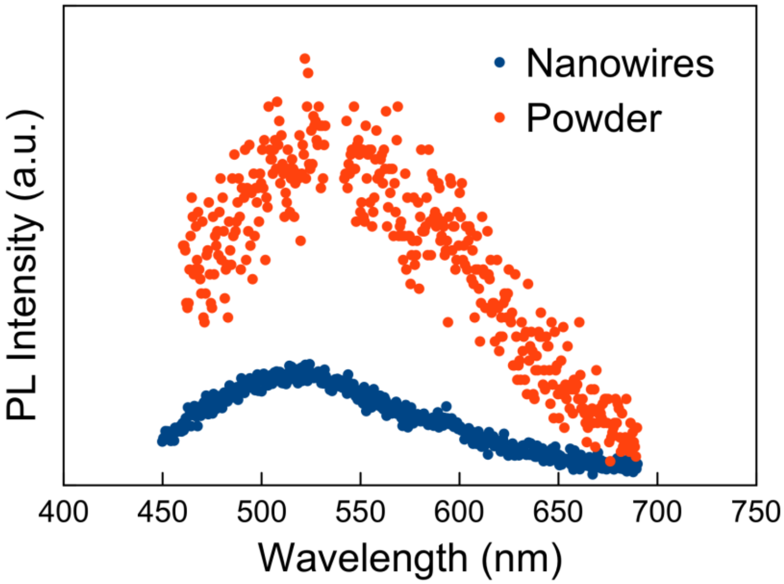

Figure 5: Photoluminescence (PL) analysis of the GaN:ZnO nanowires prepared via atmospheric pressure chemical vapor deposition, compared to those of GaN:ZnO powder synthesized through amonolysis of Ga^{3+}/Zn^{2+} layered double hydroxide.

Supplementary information 6: Variation in morphologies of the synthesized thin film

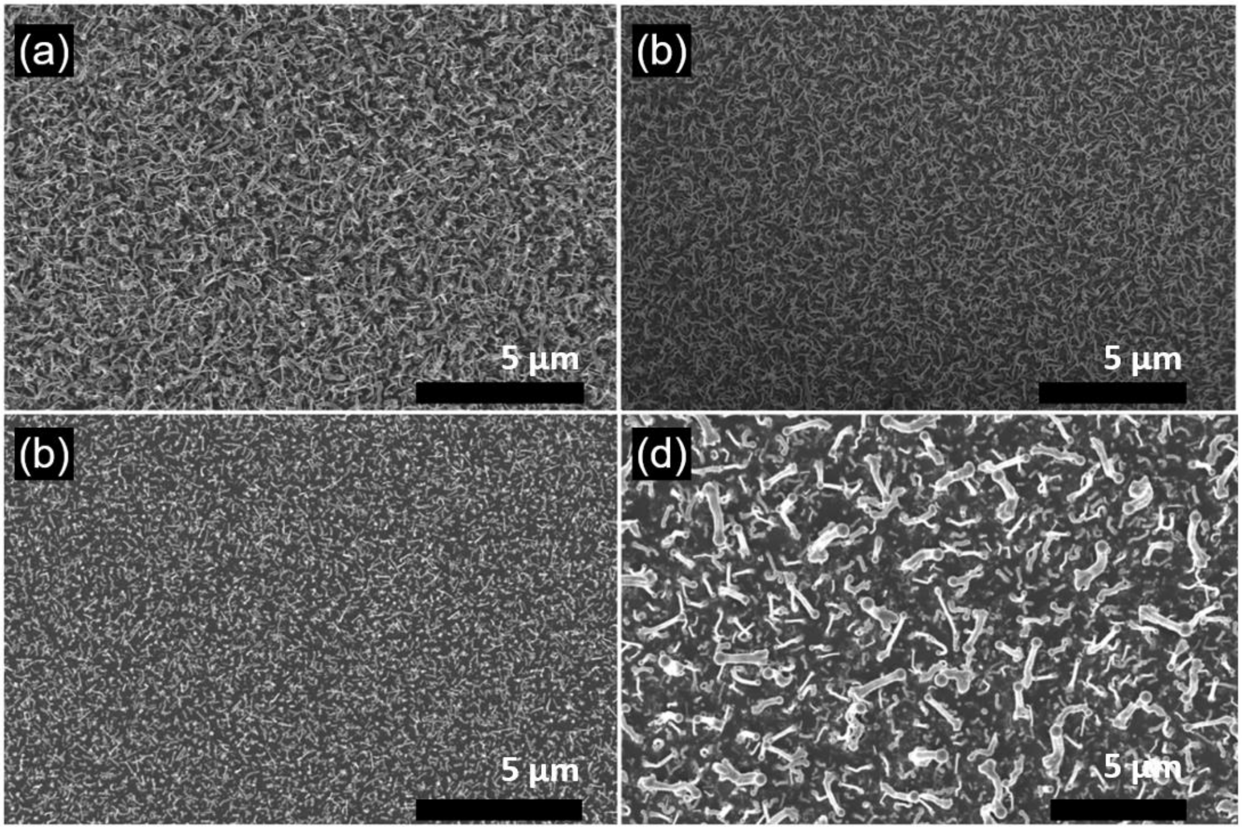

Figure 6: SEM images showing the morphology of the GaN:ZnO thin films prepared at various conditions. **a**, T_s = 1100 °C, t = 5 min, and D = 8 cm – scale bar = 20 µm. **b**, T_s = 1100 °C, t = 5 min, and D = 13 cm – scale bar = 10 µm. **c**, T_s = 1000 °C, t = 30 min, and D = 13 cm – scale bar = 50 µm. **d**, T_s = 900 °C, t = 60 min, and D = 8 cm – scale bar = 5 µm.

Supplementary information References

1. Xia, Y. *et al.* One-dimensional nanostructures: Synthesis, characterization, and applications. *Adv. Mater.* **15**, 353–389 (2003).

2. Wu, Y. *et al.* Inorganic semiconductor nanowires: rational growth, assembly, and novel properties. *Chemistry* **8**, 1260–8 (2002).
